# The Role of Meteorin-Like Peptide and Asprosin in Colon Carcinoma

**DOI:** 10.7759/cureus.47073

**Published:** 2023-10-15

**Authors:** Elif Onat, Nevin Kocaman, Hilal Balta

**Affiliations:** 1 Medical Pharmacology, Adiyaman University, Adiyaman, TUR; 2 Histology and Embryology, Firat University Faculty of Medicine, Elâzığ, TUR; 3 Pathology, Firat University, Elâzığ, TUR

**Keywords:** asprosin, meteorin-like peptide, adipokine, carcinom, colon

## Abstract

Introduction: Colon cancer is one of the most frequent gastrointestinal system cancers on a global scale. Common colonoscopy tests have reduced the incidence of colorectal cancer (CRC). Although nutrition, microorganisms, and their metabolites are related to colon cancer, the exact mechanism of CRC is still not clear. For this reason, it is of great importance to elucidate the molecular mechanisms of colon oncogenesis.

Methods: This study was conducted retrospectively with samples obtained from the laboratory of Firat University Faculty of Medicine, Department of Pathology. A total of 30 patient samples were used. The control group consisted of healthy colon tissues from the same patients, and the other group consisted of colon carcinoma tissues from the same patients. Tissue samples of both groups were evaluated immunohistochemically with meteorin-like (METRNL) peptide and Asprosin.

Results: The immunoreactivity of METRNL was found to be lower in colon carcinoma tissues than in healthy colon tissues (0.2 ± 0.06 and 0.08 ± 0.03, respectively). Asprosin immunoreactivity was found to be higher in colon carcinoma tissues than in healthy colon tissues (0.4 ± 0.07 and 1.08 ± 0.15, respectively).

Conclusion: As a result of this study, it was observed that there was a significant difference between healthy colon tissue and colon carcinoma tissue in terms of METRNL and Asprosin expression. Both proteins might be involved in the molecular mechanism of colon carcinoma. This situation is important in terms of diagnosis.

## Introduction

Colorectal cancer (CRC) is the second most frequently diagnosed cancer in women and the third in men [1]. When diagnosed at an early stage, the five-year survival rate of CRC is high, and early resection of CRC allows patients to recover close to 100% [2,3]. Therefore, predicting the probability of CRC is crucial for the prevention, early diagnosis, and appropriate treatment of CRC.

Meteorin-like peptide, which is also named METRNL, Meteorin-β, Subfatin, and Cometin, is a novel protein that was shown to have pleiotropic impacts on inflammation, metabolism, and the immune system. Studies conducted on this protein were all about the modulation of energy metabolism and glucose homeostasis. Many studies aimed at understanding the molecular mechanism of METRNL in glucose metabolism and obesity-related problems [4]. Recent studies have begun to recognize its protective effects in the regulation of inflammatory immunity and cardiometabolic disorders (e.g., stimulation of macrophage activation, vascular restructuring, tissue regeneration, bone formation, and inhibition of lipid disorders). To establish this novel protein as a biomarker in some diseases, it is important to have a better understanding of its functions and therapeutic aspects [4].

Disorders in adipose tissue disrupt adipokine secretion, resulting in many metabolic diseases (excess weight, diabetes, and cardiovascular disorders). Asprosin is a novel diabetogenic adipokine, which is released from white adipose tissue as a result of fasting and exerts glucogenic and orexigenic effects. Although white adipose tissue is not the primary source of adipokine, Asprosin can also be released from other tissues such as salivary glands, pancreatic B cells, and cartilage. Plasma Asprosin levels are associated with glucose and lipid metabolism, insulin resistance, and β-cell function. Asprosin also plays an important role in the metabolic process, stimulates hepatic glucose production, and affects appetite [5]. Clinical and pre-clinical studies have proven that there is an irregularity in circulating Asprosin levels in various cancer types and metabolic diseases such as being overweight, type 2 diabetes (T2DM), polycystic ovary syndrome (PCOS), and non-alcoholic fatty liver disease (NAFLD). Asprosin might be promising for the diagnosis of many diseases and the discovery of novel pharmacological treatment modalities, but a better understanding of its functions and signaling pathways is needed [5].

In the present study, the researchers tried to investigate the roles of these proteins in the pathogenesis of colon carcinoma by examining the expression of METRNL and Asprosin in healthy colon tissue and colon carcinoma tissue.

## Materials and methods

Research and publication ethics

The Local Ethics Committee of Firat University approved this study. The study used 30 samples, which were obtained from the laboratory of Firat University, Faculty of Medicine, Department of Pathology. The control group consisted of healthy colon tissues of the same patients. The other group consisted of colon carcinoma tissues of the same patients. All tissue samples were immunohistochemically stained with METRNL and Asprosin. The results were evaluated by making comparisons between groups.

Immunohistochemistry

The immunohistochemical procedures were performed as described by Kocaman and Artaş [6]. For immunohistochemistry (IHC), 3 µm thick histological tissue microarray slides were used. Anti-METRNL (MBS7004241; MyBioSource, San Diego, CA) and anti-Asprosin (FNab09797; Fine Test, Palm Coast, Florida) antibodies were used. The results were evaluated, and photographs were taken using the Zeiss Axio Scope A1 microscope (Carl Zeiss Microscopy GmbH, Jena, Germany). Indirect immunohistochemical staining was performed to measure the tissue levels of METRNL and Asprosin, and the histoscore was calculated.

Microscopic evaluation of staining intensity

The data were compared and evaluated by one blinded independent pathologist and one blinded independent histologist according to the extent and intensity of the staining, and then histoscoring was performed using the following criteria: 0.1 for ˂25% staining distribution; 0.4 for 26%-50%; 0.6 for 51%-75%; and 0.9 for 76%-100%. Staining intensity was as follows: 0 for no staining; 0.5 for very slight staining; 1 for little staining; 2 for moderate staining; and 3 for very strong staining.

Histoscore was calculated as follows:

$Histoscore\ =\ Distribution\times Density$ [6].

Statistical analysis

The SPSS 22 program (IBM Corp., Armonk, NY) was used for analysis. The one-way ANOVA test was used, and post-hoc multiple comparisons were made using the Tukey honestly significant difference (HSD) test. The Kolmogorov-Smirnov test was used as the normal distribution test. The data were given as mean ± SD, and p < 0.05 was considered statistically significant.

## Results

Immunohistochemical findings

The following results were obtained while examining immunohistochemical staining for METRNL and Asprosin immunoreactivity under a light microscope.

**Figure 1:**
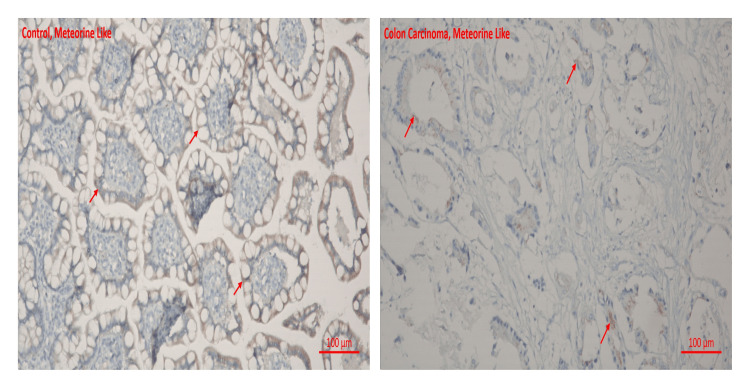
Immunohistochemical analysis of METRNL protein in colon METRNL: Meteorin-like.

METRNL immunoreactivity was found to be significantly lower in colon carcinoma tissues than in healthy colon tissues (0.2 ± 0.06, 0.08 ± 0.03, respectively) (p < 0.001) (Table 1). METRNL immunoreactivity histoscores of the groups are given in Figure 1.

**Table 1:** METRNL and Asprosin immunoreactivity histoscore Values are given as mean ± standard deviation. ^a ^Compared with control (p < 0.001). METRNL: Meteorin-like.

Groups	Control	Colon carcinoma
METRNL	0.2 ± 0.06	0.08 ± 0.03^a^
Asprosin	0.4 ± 0.07	1.08 ± 0.15^a^

**Figure 2:**
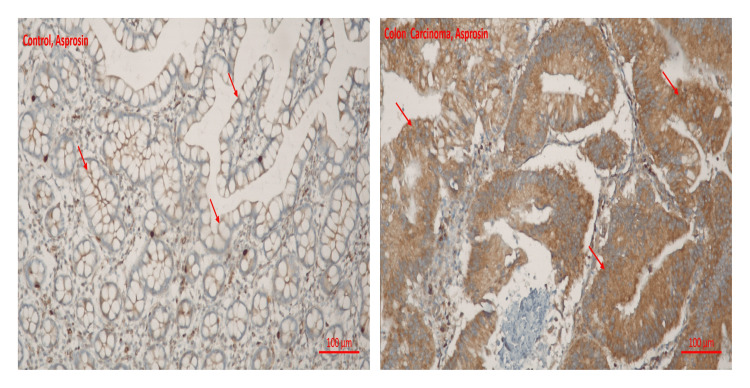
Immunohistochemical analysis of Asprosin protein in colon METRNL: Meteorin-like.

Asprosin immunoreactivity was found to be significantly higher in colon carcinoma tissues than in healthy colon tissues (0.4 ± 0.07, 1.08 ± 0.15, respectively) (p < 0.001) (Table 1). Asprosin immunoreactivity histoscores of the groups are given in Figure 2.

## Discussion

Colon carcinoma is among the most common and aggressive tumors worldwide. Although the morbidity and mortality of colon carcinoma have tended to decrease slightly over the last decade, it remains the main cause of cancer-related deaths [7,8]. For this reason, new studies are needed for the diagnosis and treatment of colon carcinoma. In the present study, the researchers found a significant difference in terms of METRNL and Asprosin expression between healthy colon tissue and colon carcinoma tissue and thought that these proteins might have diagnostic and treatment potential.

Research results show that many adipokines might affect tumor formation and cancer progression over cell migration and proliferation and increase anti-apoptotic pathways [9]. A previous study reported that low serum METRNL levels might be associated with endothelial dysfunction [10]. For this reason, it is considered that METRNL expression might play a role in regulating the function of colorectal endothelial cells. It was reported in another study that METRNL was expressed at high levels in early embryos during gastrulation and was very important for mesoendoderm development [11]. These also add to the evidence that METRNL regulates the function of colorectal endothelial cells. In conclusion, METRN overexpression is considered to be closely associated with advanced CRC stages and poor clinical outcomes. This might have a reference value for the prognosis of CRC patients in future clinical applications [12]. In the present study, the researchers observed that METRNL was lower in colon carcinoma tissue than in healthy colon tissue. If literature studies are taken as a reference, it is possible to argue that this is associated with the early stage of the disease or the stable clinical condition of patients. However, to make this statement, it might be necessary to consider other clinical characteristics of patients. Furthermore, as METRNL is a novel molecule with limited knowledge, it would be premature to make a definitive comment at this stage.

Little data is available on Asprosin levels in carcinomas. In a study, it was discovered that the expression levels of Asprosin and its conjugated olfactory receptor OR4M1 were increased in healthy and cancerous human ovarian tissues. In this way, they showed their effects in the tumor environment [13]. In another study from the same group, it was determined that there was a difference in the regulation of genes after 100 nM Asprosin treatment of the ovarian cancer cell line SKOV-3 [14]. Asprosin also stimulates ERK1/2 phosphorylation, which is associated with cell communication and proliferation. Asprosin altered many signaling pathways associated with cell communication and proliferation by stimulating ERK1/2 phosphorylation [14]. However, in a study in which reactive mesothelial hyperplasia (RMH) samples were considered as a control group and compared with the surface tumor known as malignant mesothelioma (MM), Kocaman and Artaş found that there was an increase in the Asprosin expression and immunoreactivity [6]. Another study by Kocaman et al. showed the possibility of using Asprosin in the differential diagnosis of two skin cancer types stemming from hair follicles called basal cell carcinoma (BCC) and hair follicle trichoblastoma [15]. They observed high Asprosin immunoreactivity in BCC samples, while there was no change in trichoblastoma samples [15]. Asprosin is known to change the antioxidant-oxidant balance by causing hyperinsulinemia and insulin resistance, thereby increasing the expression level of insulin-like growth factor 1 (IGF-1) [16,17], which causes cancer formation and metastasis [18]. In the present study, the increased expression of Asprosin in colon carcinoma tissue compared to healthy colon tissue supports the results of previous studies conducted on Asprosin so far. Asprosin appears to be a potential target molecule for the diagnosis and treatment of colon carcinoma. However, more studies are needed in this field.

The most important limitation of the present study was that it did not address other clinical characteristics of the patients and had a retrospective design. More detailed studies involving a larger number of patients are needed because METRNL and Asprosin are very novel molecules.

## Conclusions

The results of the study support the presence of a significant relationship between healthy colon tissue and colon carcinoma tissue in terms of METRNL and Asprosin expression. Both proteins might be involved in the molecular mechanism of colon carcinoma. As a result, the increase in METRNL and Asprosin in colon carcinoma suggests that these proteins may be therapeutic targets in the diagnosis and treatment of colon carcinoma, one of the most common cancers. These molecules may form the basis for further research.
